# Supplementary figures and images for: Drosophila-associated bacteria differentially shape the nutritional requirements of their host during juvenile growth
Source: PLoS Biol. 2020 Mar 20;18(3):e3000681. doi: 10.1371/journal.pbio.3000681 (PMC7112240; doi:10.1371/journal.pbio.3000681)

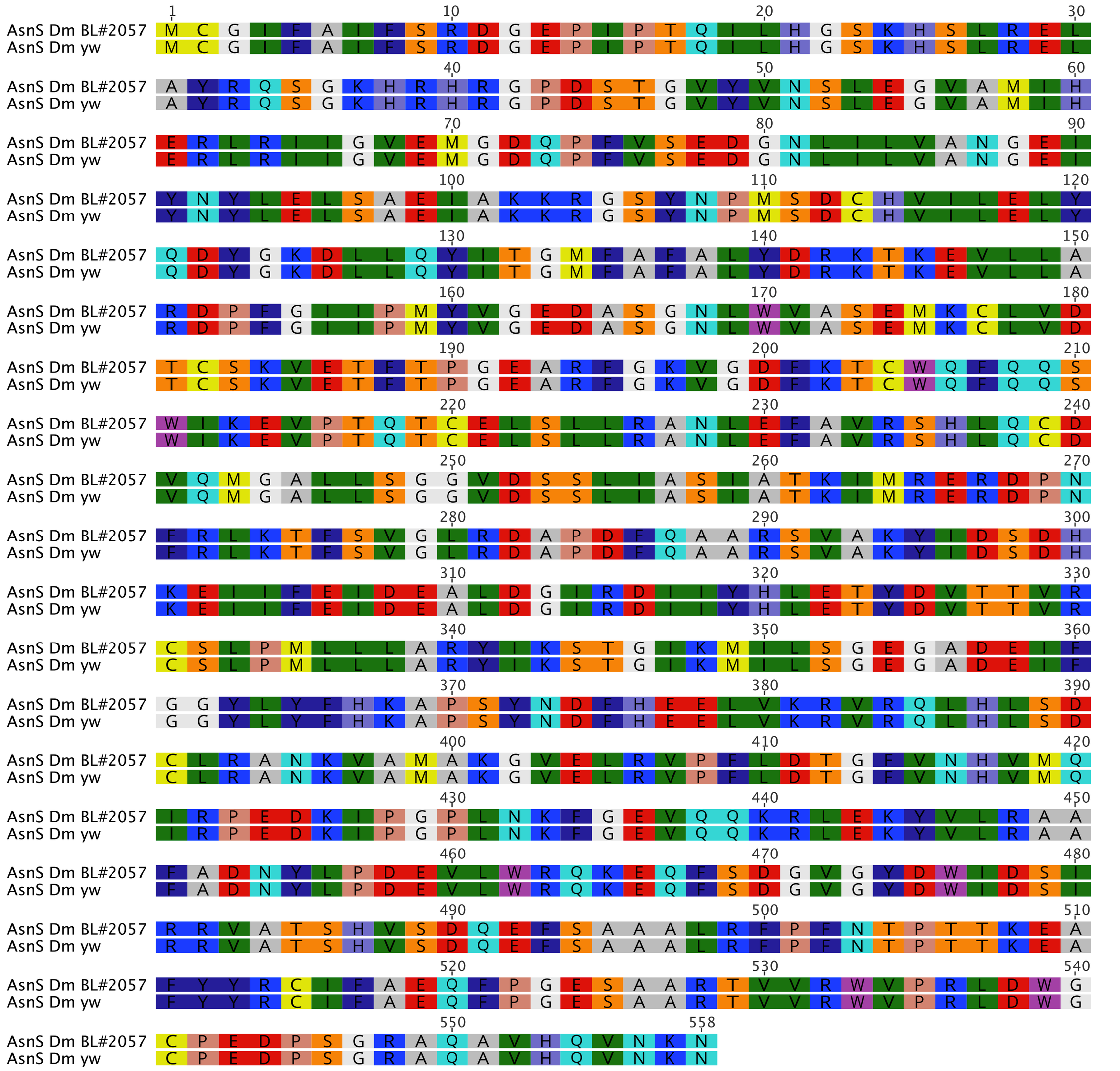

Supplement: S1 Fig — Pairwise alignment of the AsnS coding region sequenced from D. melanogaster yw and the AsnS coding region from D. melanogaster reference genome, Bloomington #2057. yw, yellow-white. (TIF) [file pbio.3000681.s001.tif]

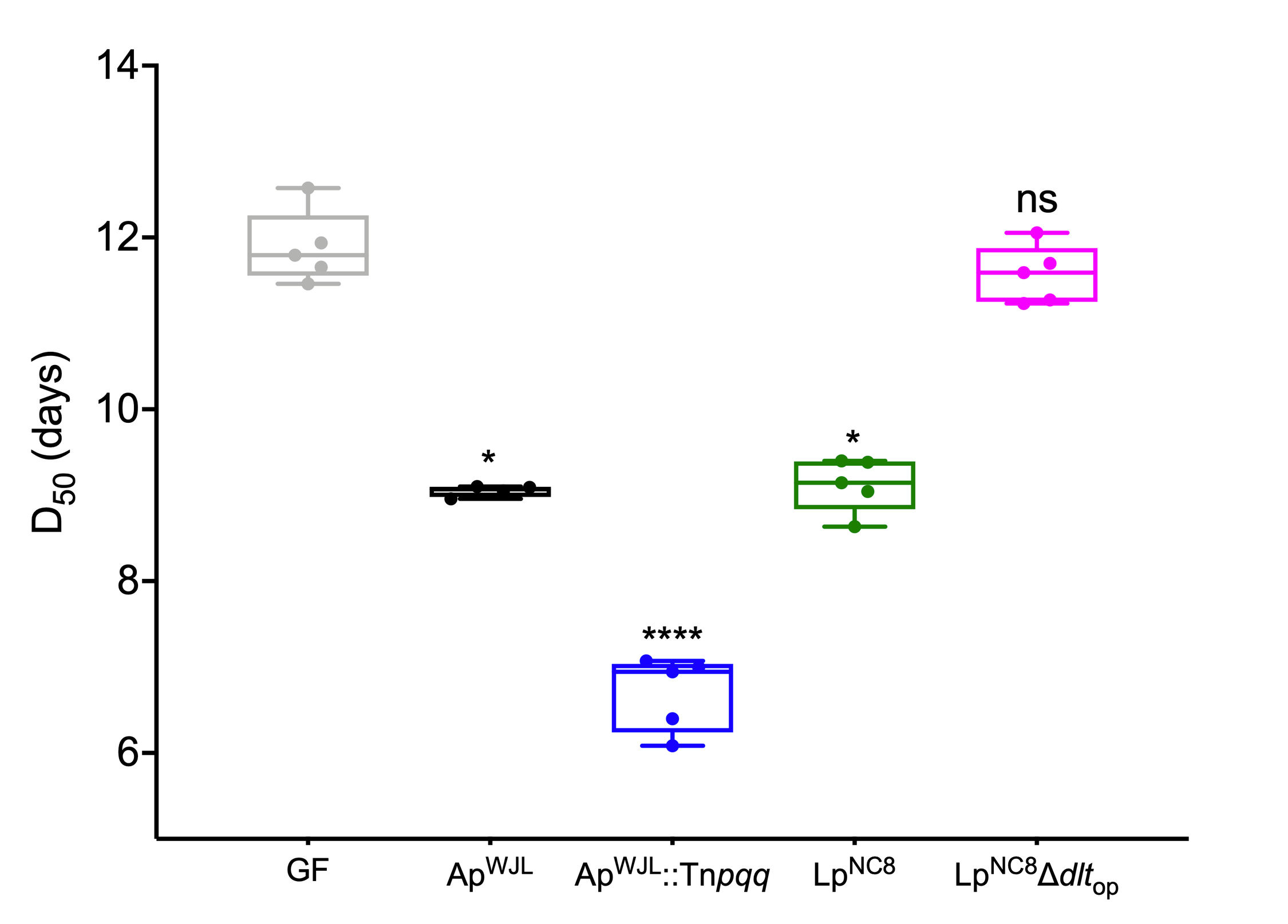

Supplement: S2 Fig — D50 of GF larvae and larvae associated with ApWJL, ApWJL::Tnpqq, LpNC8, and LpNC8Δdltop, reared on complete HD. We performed a Kruskal–Wallis test followed by post hoc Dunn tests to compare each gnotobiotic condition to GF. *p-value < 0.05, ****p-value < 0.0001. ApWJL, A. pomorumWJL; dlt, XXX; D50, day when 50% of larvae population has entered metamorphosis; GF, germ-free; HD, Holidic Diet; LpNC8, L. plantarumNC8; ns, nonsignificant; pqq, pyrroloquinoline-quinone–dependent; Tn, transposon. (TIF) [file pbio.3000681.s002.tif]

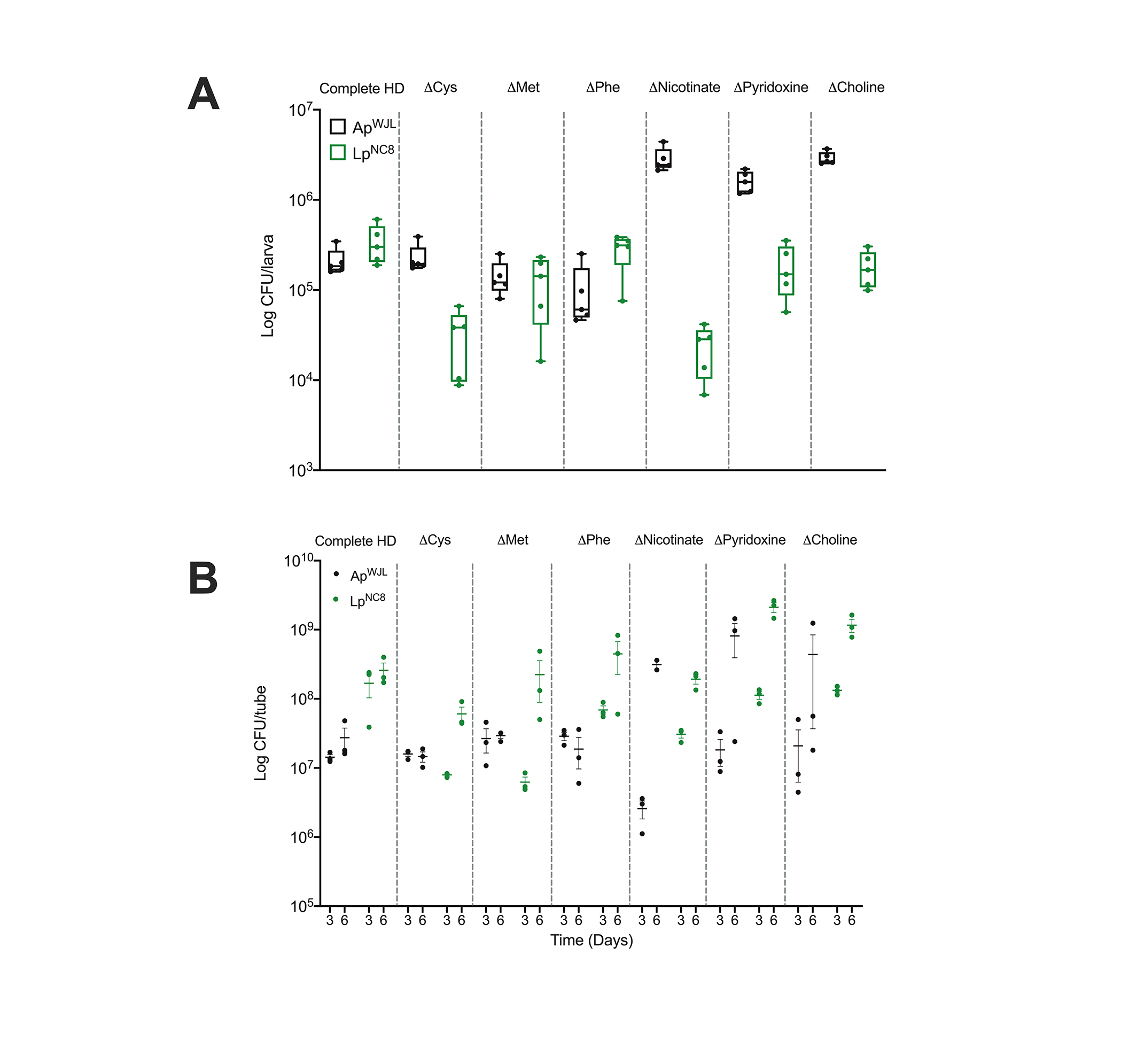

Supplement: S3 Fig — (A) Bacterial load per larva at day 6 postinoculation. Boxplots show minimum, maximum, and median. Each dot shows an independent replicate. (B) Load of ApWJL and LpNC8 in solid HD in presence of larvae 3 days and 6 days after inoculation. Plot shows mean with standard error based on 3 replicates by assay. Each dot represents an independent replicate. The dashed line represents the level of inoculation at t = 0 h (104 CFUs per tube). ApWJL, A. pomorumWJL; CFU, colony-forming unit; HD, Holidic Diet; LpNC8, L. plantarumNC8. (TIF) [file pbio.3000681.s003.tif]
