# Supplementary material for: Drosophila-associated bacteria differentially shape the nutritional requirements of their host during juvenile growth
Source: PLoS Biol. 2020 Mar 20;18(3):e3000681. doi: 10.1371/journal.pbio.3000681 (PMC7112240; doi:10.1371/journal.pbio.3000681)
Supplement: Attachment — Submitted filename: Point-by-point_answers.docx [file pbio.3000681.s011.docx]

**Point-by-point response to the reviewer and editor comments on the PBIOLOGY-S-19-02724 manuscript entitled “*Commensal bacteria differentially shape the nutritional requirements of Drosophila during juvenile growth.”***

We would like to thank the editor and reviewers for their helpful comments and constructive criticisms. We believe that we have now addressed their concerns and we think that our revised text and our new results have improved the manuscript.

Briefly, we have now significantly edited our manuscript to convey our results and interpretations in a simpler and more focused manner. We provide new experimental data demonstrating that in relevant settings Lp^NC8^ and/or Ap^WJL^ do not synthetize choline, pyridoxine or nicotinic acid yet they sustain development of their host on diets lacking these nutrients supporting the idea that the commensals can functionally compensate for the lack of an essential nutrient. Moreover, we provide further experimental data establishing the absence of correlation between bacterial load in the larval gut or in the diet and the host developmental timing on the tested diets. These results reinforce the notion that commensal bacteria are not “just food” for their animal partner but are biologically active partner to their host.

Please find below a point-by-point answer to the reviewers and the editor’s comments (copied in bold below).

**Reviewer #1:**

**This manuscript by Consuegra and colleagues describes a detailed and thorough set of experiments that define the nutrient requirements of the fruit fly, Drosophila melanogaster, and two of its commensal bacteria, Acetobacter pomorum and Lactobacillus plantarum. They match in silico analyses of the biosynthetic capabilities of each organism with in depth experiments exploring the growth capacities of each bacteria, and of Drosophila with and without bacteria on a chemically defined (holidic) diet. This allows them to explicitly address how the nutrient requirements can be met by bacteria under nutrient stress.**

**By sequentially dropping out each of the 38 nutrients in the holidic diet, they first determine the precise nutrient requirements for growth of both bacteria and for egg to pupal development in Drosophila. Next, they found that when they co-culture larvae with either bacteria on these drop-out diets, the bacteria are able to rescue larval development for a limited set of nutrients that are essential to larvae. These commensals differ in their ability to compensate, with A. pomorum able to sustain larval development over a greater number of nutrient-depleted diets. The authors supplement these findings by feeding heat killed bacteria to larvae on the holidic drop-out diets. Heat killed bacteria were unable to rescue developmental timing for any of the fly essential amino acid drop-out diets. This eliminates the potential that the observed effects are due to the fact that the bacteria themselves are being used as food.**

**Finally, the authors conduct experiments to try to understand how the commensal bacteria supply essential nutrients to their host. They do this by culturing bacteria in the drop-out diets for 18/22 amino acids and assessing whether the bacteria secrete the missing amino acid. Curiously, while bacteria rescue developmental timing in larvae for a range of amino-acid drop-out diets (all 22 amino acids for A. pomorum, 15 amino acids for L. plantarum), they were only able to detect 7 and 1 amino acid in the supernatant for A. pomorum and L. plantarum respectively. This highlights that future work will be key to understanding how bacteria deliver the necessary nutrients to their host**.

**This work demonstrates in an exquisitely precise manner the relationship between a host, it’s microbes, and the host’s diets. This type of in depth analysis is possible in only a few organisms, but will serve for generating broad perspectives on host/commensal interactions under dietary stress. Given that the microbiome has been linked to growth, fitness, and health of a wide range of animals, this work is sure to be of interest to biologists and health scientists**.

**I find this work to be very thorough, and the authors have taken care to use appropriate statistical methods. Further, their story is clear and well-explained. I have only a few comments that I think might help**.

We thank the referee for her positive assessment of our work.

**In the results section (lines 369-371), it would be useful to briefly outline the rearing conditions of the larvae (especially because the methods come after the results). I was unsure, until I checked the methods, whether these larvae were reared from egg until pupae on the holidic diets.**

The information has now been added page 15, line 347.

**The authors mention that they were unable to detect a number of amino acids in the supernatant when their bacteria were cultured on the holidic diet, and discuss potential reasons why. This was especially notable in L. plantarum, where they were only able to detect the secretion of histidine. I think it would be worth raising the point that the culture conditions of bacteria on holidic diet are likely to differ significantly from the conditions in the larval guts, and that both A. pomorum and L. plantarum could very well be receiving cues from the larva itself to produce/secrete these nutrients**.

We fully agree with the referee’s hypothesis and have now included this point to our discussion of the result (page 28, line 667-669).

**Minor comments:**

**Line 68: The abbreviation “Dm” is used infrequently throughout the text. My preference would be to use "Drosophila", to avoid introducing additional acronyms. Also, in some parts you use Drosophila, others D. melanogaster. For consistency, I would pick one.**

We agree with the referee and we are now mostly using “*Drosophila*” all along our manuscript when we reference to the host. We only use *D. melanogaster* when referring precisely to the genome or a given fly strain.

**Line 317: Consider changing “On the contrary” to “In contrast”.**

The change has been made.

**---------------**

**Reviewer #2:**

**The study by Consuegara and Greiner et al. looks at the potential of two major gut commensals of Drosophila melanogaster (Dm) to modulate the nutritional requirements of the host.**

**It is well known that the gut microbiota plays an important role in nutrition of the host, in particular in the case of malnutrition and during juvenile growth. However, a systematic experimental study to identify which dietary nutrients can be complemented by a gut symbiont has not been conducted. In the current study, the authors first predict - based on genomic data and in silico metabolic network reconstruction - the biosynthetic capacities of Dm germ-free larvae and the two major gut commensals. Then, they carry out an elegant nutritional screen to experimentally determine which nutrients of a defined larval diet can be complemented by the two gut commensals to sustain larval growth.**

**To the best of my knowledge, there is no other study that has conducted a similar systemic screen to probe which nutritional requirements of the host can be complemented by gut commensals. The results provide many new insights into the nutritional roles of the two major gut commensals of Dm and show that the metabolism of the host and the gut commensals are highly integrated. Of particular interest is the combination of in silico predictions and experimental data, because this combined approach allowed the authors to discovery interactions that otherwise would not have been identified.**

**Overall this is an exciting study with many new and relevant insights for the field of microbiome research. The experiments seem to have been designed and carried out with great care and the results are well presented in both text and figures. My only two major points are that the manuscript seems to be very long and the language sometimes more complicated than needed.**

We thank the referee for the positive assessment of our work. We have now significantly edited our manuscript to convey our results and interpretations in a simpler and more focused manner.

**Major comments:**

**Language could be simplified. The broad readership of PLoS Biology will have difficulties to fully appreciate the relevance of this paper. In particular, phrasing of the abstract is overly complicated. A striking example is the first sentence. Wouldn’t it be as simple as writing “The interplay between nutrition and the gut microbiome plays a central role in determining juvenile growth in animals”? I would recommend to carefully go through the entire manuscript and remove jargon and unnecessarily complicated phrases.**

We have now revised our text, and we feel we have removed most jargon and complex sentences.

**I would also recommend to replace the term ‘microbial environment’ with something else throughout the text, because this term could also refer to the environment of the bacteria instead of the bacterial characteristics of the host environment. Why not use ‘composition of the microbiome’ or simply ‘microbiome’ or ‘host-associated microbiome’?**

We now avoid using the term “microbial environment” and use “Host-associated microbes” in few instances to avoid confusion (page 4, lines 72-75).

**2. My second major concern is that the manuscript seems to be overly long. It is fine that authors take great care in explaining and discussing their results. However, in particular the first section of the Results/Discussion about the metabolic capabilities of the three partners is lengthy (Line 169-265). Most of it could be moved to the supplementary, and authors could simply keep the last paragraph of each section which summarizes each partners’ capabilities as based on the analysed genomic data. I would also recommend that the authors make an effort to go through the other sections and try to shorten to keep the focus on the most relevant findings.**

We have shortened the description of the metabolic capabilities of the 3 partners and only kept what we feel was strictly necessary to this study (pages 8-14, lines 150-235) and have also tried to condense our manuscript as much as possible in other places.

**Minor:**

**3. I would not use the word semi-essential, but rather consistently use 'conditionally essential'**

The change has been made (page 3, lines 44 and 47).

**4. Line 50. Instead of ‘transformed’, ‘modulated’ may be a better term.**

The change has been made (page 3, line 49).

**5. Line 99: May be this is just me, but I do not understand why the finding that the bacteria-mediated growth benefits are only observed under nutrient scarcity would suggest that the microbiota complements nutrients.**

**Previously, the authors and others have shown that bacteria-mediated growth benefits are due to the induction of peptidases and insulin signalling. Can’t these effects be sufficient to explain the beneficial growth? May be provide a better explanation.**

We have now adjusted the explanation of our rationale (page 5, lines 96-101). Indeed, it was previously shown that dietary protein digestion is enhanced upon microbial association. However, such biological phenomena alone would not compensate for the scarcity of essential nutrients in a globally poor diet. Therefore, we postulated the existence of microbe-mediated compensation of essential nutrients that cannot be compensated by increased protein digestion; substantiating this phenomenon is the focus of our manuscript.

**6. Line 146: I disagree that this is a ‘unique’ opportunity. Genomes of many gut symbionts and their dedicated hosts are available allowing similar studies**.

Indeed, this is an overstatement; we have now changed this part of the introduction (page 7, lines 142-148).

**7. Fig 3B. ‘Growth x-times inoculum’ is a strange way of plotting the data and labelling the axis. Why not showing absolute number of CFUs, e.g before and after growth? Authors state that there was no difference in growth between conditions, but no statistics have been applied. This should be revised.**

We have changed the labeling of the new Fig 3B, Fig 5B-C-D and Fig S3A-B. We have added a dashed line to illustrate the inoculum when appropriate and instead of “growth x-times inoculum” we use “CFUs/mL, CFUs/Tube or CFUs/larvae” to illustrate bacterial loads as in the other panels of the paper.

**8. Fig 4A. Why does this heatmap not go from dark red to white as in the previous figure? A heatmap from red to yellow, to green, and to blue makes little sense, and at least to me it is not intuitive which color corresponds to long or short developing time, especially as ‘no development’ is assigned the ‘color’ white.**

While preparing this heat-map representation we had tested multiple versions of it using different color sets. Visually the one we adopted was the most illustrative especially given that we always include the legend next to the heat-map. Using the red to white color range was not discriminative enough for the developmental timing data sets as we needed to discriminate among the following conditions: fast development (red), slow development (green), viable development but extremely delayed (blue), no development/lethality (white). This is in contrast with bacterial growth data, which can be fully captured with a simple growth (red) vs no growth (white) range. We therefore prefer to keep this representation.

**9. Line 403. A less cryptic section title would be helpful**.

We have now changed this section title to “Bacterial cell walls sensing contributes to Lp^NC8^ mediated larval growth promotion in complete chemically defined diets” (page 17, line 385-386).

**10. Line 444. The title of this section also needs to be edited in my opinion. I would suggest writing ‘Fulfills … requirements’. I don’t think ‘Fulfills … auxotrophies’ is what the authors mean here.**

We agree with the referee’s comment and we have now adjusted this (page 19, lines 427 and 436).

**11. Line 573: A more conceptually description of case 4 would be desired. I would also replace the word ‘case’ by ‘scenario’ for all four descriptions. As these are not individual cases (like case studies…), but possible types of nutrient complementation**.

We have now changed “case” by “situation” and further described situation 4 (page 26, lines 599-628). We also provide a new title this subsection: “Situation 4: Bacterial compensation of minerals and metal deficiencies by concentrating traces or by functional compensation” (page 26, lines 599-600).

**12. The discussion of the four different ‘cases’/’scenarios’ is interesting. However, it would be good to know how many of the tested nutrients belong to each of these scenarios. Which one is the dominant type?**

We have amended our sub-section headings (pages 21, 22 and 26, lines 483, 517 and 599) to include how many tested nutrients belong to each situation. We have also amended the conclusion paragraph accordingly; we now state “The capacity of the bacteria to fulfill 19 of the requirements in 22 essential nutrients for larvae correlated with their metabolic activity and in most cases (15 out of 19) their ability to produce the missing nutrient.” (page 30, lines 695-698).

**13. Line 673. Very long sentence. Why not split in two?**

We changed this (page 30-31, lines 708-713).

**14. Line 686. Instead of ‘foundational blueprint’, why not simply say ‘basis’? These and other complicated words should be removed to keep the text less cryptic. (see my point 1)**

We changed this (page 31, line 724).

**15. To colonize germ-free Dm, bacteria were inoculated on the growth substrate and the Dm eggs. To test the effect of heat-killed cells, 10x more heat-killed bacterial cells than live bacterial cells were inoculated. This sounds like a lot. However, it is not clear how much the live bacteria grew after inoculation on the substrate. It may be much more than 10x, and hence the bacteria could simply serve as ‘food’ rather than active symbiotic partners for nutrition. Could the authors please comment on this?**

**Likewise, it would be interesting to know more about the bacterial abundance in the gut? Did both bacteria colonize the gut to the same extent under the different nutrient conditions tested that may have influenced their capacity to complement nutrients.**

We have now added new data and discussed this point in our revised manuscript (page 20-21, lines 460-473; page 31, lines 714-720).

In a previous paper from our lab (Storelli et al. 2018), we showed (see Fig.4 of this paper) that, when using a high inoculation load (i.e., an experimental context where the bacteria do not increase their load in the fly food over time), inoculating germ-free larvae with the same amount of heat-inactivated bacteria does not confer growth promotion to the larvae. In this context, it is only upon repeated (3 times) inoculation of 100X excess heat-inactivated bacteria that we could detect a small but significant effect on larval growth. However, this effect does not mimic the effect of a single 100X excess alive bacterial inoculation. This result establishes that an excess of dead bacteria can indeed have a small but significant trophic effect on the host (like providing more dead yeast) but that alive bacteria do confer an added benefit to their host as compared to the addition of mere “food” in the form of dead bacteria. Of note, alive yeast cells are also more effective at promoting growth than dead yeast. An observation also reported with other microbes by the Ja lab (Yamada, Keebaugh 2018,2019). This increased impact of living micro-organisms on their host’s development is the biological phenomena that we are interested in and that we and others have been studying in the past (Shin et al. 2011, Erkosar et al. 2015; Matos et al. 2017; Martino et al. 2018). Beyond these first studies using complex oligidic diets, we are now using holidic diets to reveal the whole complement of essential nutrients that bacteria can functionally compensate, synthesize, or spare to their animal partner. In the revised version of the manuscript, we are now citing the previous observations that dead microbe can provision macronutrients to *Drosophila* (page 31, lines 715-716).

Referees #3 and #4 also commented on this point, while Referee #1 stated “Heat killed bacteria were unable to rescue developmental timing for any of the fly essential amino acid drop-out diets. This eliminates the potential that the observed effects are due to the fact that the bacteria themselves are being used as food”.

Therefore we agree with the editor that it is important to tackle again in our setting the question “does the bacteria simply serve as ‘food’ rather than active symbiotic partners” experimentally. To this end, we ran additional experiments in specific holidic diets set-ups. We assessed the potential correlation between bacterial loads and host growth benefits upon different nutrient drop out in contexts, where both (Full HD) or each differently (HDΔMet, HDΔPhe, HDΔNicotinate) or only one (HDΔCys, HDΔPyr, HDΔCholine) of the bacteria would impact host growth. As shown in the new Figures 4B-D and Fig S3A-B, we found no correlation between bacterial load in the larval gut or in the diet and the host developmental timing on the tested diets. These results again reinforce the notion that commensal bacteria are not “just food” for their animal partner but are also biologically active partner to their host. We are now presenting and discussing these new results in the revised version of the manuscript (pages 20-21, lines 460-473).

---------------

**Reviewer #3:**

**The study by Consuegra and coworkers addresses metabolic crosstalk between Drosophila melanogaster and its two commensal bacteria, Lactobacillus plantarum and Acetobacter pomorum. The authors use in silico metabolic network reconstruction to predict the biosynthetic capacities of Drosophila melanogaster and the two commensals. The authors then systematically test the metabolic dependencies of specific nutrients by utilizing a set of 40 different fully defined diets, lacking one nutrient at a time. In general, the research question is valid and experiments are rigorously conducted.**

**The data obtained in experiments with holidic diets is in good agreement with the in silico reconstruction: germ free Drosophila melanogaster larvae are auxotrophic for nutrients that cannot be synthesized by it and these auxotrophies are compensated by the commensal, in case it has an intact biosynthetic pathway to synthesize the nutrient. In most cases, bacteria need to be living to compensate for the missing nutrient, although some exceptions are found. Moreover, the study uncovers that Drosophila melanogaster and its facultative commensals have separated metabolic pathways, in contrast to earlier findings on obligate symbiosis. While these findings are not particularly surprising, the systematic nature of this study makes it an important reference within its field. The reconstructed pathways serve as valuable resources for anyone working on metabolism of Drosophila melanogaster or its commensal bacteria.**

We thank the referee for the positive assessment of our work and for highlighting the value for the community of our *Drosophila* genome-based metabolic pathways reconstruction.

**More surprisingly, the authors find a small number of cases, where the commensal compensates for the host auxotrophy, despite the lack of a (in silico predicted) biosynthetic pathway for the nutrient. However, these potentially interesting cases remain to be further experimentally explored by the authors.**

We have now further explored these cases by performing additional genome analysis and experiments (see below).

**Although I certainly see the value of this systematic study for the scientists in this specific field, I do not currently find sufficiently strong novel biological insight to recommend publication in a general readership journal, like PLoS Biology. In my view, the study would be perhaps more suitable for a field-specific journal in its current form. However, further understanding and experimental analysis of the cases, where auxotrophy is compensated without a known biosynthetic pathway, would have the potential to make the conclusions significantly more interesting to a broader audience**.

We believe that we have addressed the issue raised by the referee by providing additional genome analysis and experimental data demonstrating that in relevant settings Lp^NC8^ and/or Ap^WJL^ do not synthetize pyridoxine or nicotinic acid but rather compensate for these missing nutrient by providing metabolic intermediates or nutrient derivatives (see details below).

**Major comments:**

**1. The authors state: “In the case of pyridoxine, ApWJL may fulfill larval requirements by either a functional compensation or through pyridoxine biosynthesis by non-canonical pathways (see above). We reach the same conclusion regarding nicotinate.”**

**The bacteria must be able to synthesize nicotinate somehow. This needs to be resolved prior to publication.**

Using ultra-sensitive LC/MS technologies, we have been able to exclude experimentally the hypothesis that Ap^WJL^ produces nicotinate. However, our genome-based metabolic predictions suggest that Ap^WJL^ may compensate for the lack of nicotinate by producing intermediates such as nicotinate-D-ribonucleotide, NAD^+^ and NADP^+^. We have added these results and hypotheses to our revised manuscript (page 23, lines 533-537). Due to technical, financial and timing issues, we did not manage to analyze all the nicotinate biosynthetic pathway intermediates and derivatives using ultra-sensitive LC/MC technologies in the context of the revision of this manuscript. However in a related project using another *Acetobacter* strain (*Acetobacter* *fabarum*^DsW_054^ from the Newell lab), we have performed an untargeted metabolomics analysis of a culture of this strain in a complete HD. Interestingly, we found a significant production of nicotinamide and NAD+ in this context (compounds that are lacking in the diet used); an observation that we feel strengthen the hypothesis we are putting forward in our revised manuscript that suggests that Ap^WJL^ may compensate for the lack of nicotinate by producing intermediates (page 23, lines 553-555). We prefer at this stage to not mention these unpublished observations in our revised manuscript but we are providing them to the referees as Fig. R1 below.

|  | ***A. fabarum* on complete HD/sterile complete HD (fold-change)** |
| --- | --- |
| Quinolinate | 1,31 |
| nicotinate ribonucleoside | 1,00 |
| Nicotinamide | 14,72 |
| nicotinamide ribonucleotide (NMN) | 1,00 |
| nicotinamide riboside | 1,80 |
| nicotinamide adenine dinucleotide (NAD+) | 2,35 |
| trigonelline (N'-methylnicotinate) | 1,19 |

**Fig. R1 Mass spectrometry measurements of nicotinate derivatives.** The table shows the ratio between quantity detected in liquid HD supernatants after 72h growth of *A. fabarum* and quantity detected in sterile HD. Note the nicotinamide and NAD+ accumulation.

**Some bacteria are able to synthesize nicotinate from both tryptophan, in addition aspartate (e.g. PMID:14700627). Are you sure both pathways are missing from both commensal strains?**

Following the reviewer’s suggestion, we have produced a deeper genome analysis related to the nicotinate biosynthetic pathway.

The first two steps of the pathway seem lacking in both bacteria, as the EC 1.4.3.16 and 2.5.1.72 are not present. We found that the succinate dehydrogenase (EC 1.3.5.1) can alternatively use oxygen or fumarate as O-donor depending on aerobic or anaerobic living conditions. Hence, this enzyme can switch between its aerobic 1.3.5.1 activity towards the 1.4.3.16 activity in anaerobic conditions using fumarate as substrate and producing imminoaspartate.

In Ap^WJL^, three candidate genes exhibit this same enzymatic activity (peg.1231, peg.1229 and peg.1228). Assuming that one of them can produce the activity at a sufficient rate in aerobic condition in Ap^WJL^, then the bacteria would be able to produce NAD and NADP from Asp. This is consistent with the fact that the salvage pathway permitting the interconversions between nicotinate and nicotinamide is highly degraded: conversely to Lp^NC8^, Ap^WJL^ does not import nicotinate, nicotinamide or nicotinate-D-ribonucleoside from the diet. This is also coherent with the growth of the bacteria in Δnicotinate diet but with a slow growth rate in agitated (aerobic) conditions, as well as with the rescue of the GF-larvae in the same deficient diet. The Figure 1B, Table S2 and text (pages 9-10, lines 181-202) have been corrected consequently.

In Lp^NC8^, one gene (nc8_0979) encodes the 1.3.5.1 activity and is a putative candidate. However, despite our efforts, we did not find any gene encoding the quinolate synthase activity (2.5.1.72, encoded by nadA gene). Moreover, the third step of the pathway (2.4.2.19, nicotinate-nucleotide diphosphorylase) is described in our database with a very poorly supported annotation (only referred by Blast2GO-EC as a secondary activity). Hence, accordingly to the work of Martino et al. 2016, the conventional metabolic route leading to NAD+ from aspartate is likely non-functional and the bacteria must import nicotinate or nicotinate–D-ribonucleotide to produce the NAD+ and NADP+. It is to note that, in Lp^NC8^, the salvage nicotinate cycle is complete. As Lp^NC8^ strain shows some growth in Δnicotinate diet and produces rescue of GF-larvae in the same diet, we investigated the possible contribution of new metabolic pathways. Hence, the Tryptophan pathway leading to quinolinate in 5 steps is the conventional eukaryotic route, but it has been described in several bacteria. Unfortunately, no significant blast hits were found for any of the five steps of the metabolic route (nor in Ap^WJL^). Again, future comparative transcriptomic analyses of bacteria grown on complete versus Δnicotinate media, combined with analysis of mutants are expected to help the identification of new candidates. Nevertheless, we consider that this work is out of the scope of the paper. The revised Fig. 1B and table S2 include the alternative routes to synthetize nicotinate (including the Trp pathway) and the text has been modified accordingly (page 10, lines 220-228).

**If yes, discovery of a new nicotinate biosynthesis pathway would certainly increase the scientific impact of the study.**

**Using genetic screening in the commensals one should be able to identify genes differentially affecting bacterial growth on Nicotinate ± media.**

We agree with the referee that this is an exciting follow-up of our study but we also agree with the academic editor that screening to find new genes involved in nicotinate and pyridoxine biosynthesis pathways is outside the scope of this paper. Also we excluded experimentally that nicotinate can be produced however nicotinate intermediates still may.

**Nicotinate (or its downstream metabolites) should be measured in nicotinate-deficient media to test the existence of biosynthesis.**

Nicotinate biosynthesis has now been experimentally excluded; this new result is included in the text (page 23, lines 533-537).

**2. The authors state: “ApWJL may synthesize other compounds that Drosophila can use to replace choline. As stated before, Acetobacter mutants precluding phosphatidylcholine (PC) synthesis shift their membrane composition towards increased content of phosphatidylethanolamine (PE) and phosphatidylglycerol (PG)… We posit that ex-GF larvae growing on HDDCholine may capitalize on ethanolamine or glycerol produced by ApWJL to compensate for the lack of choline in their diet.”**

**This is an interesting suggestion that can be tested by lipidomics. Experimental evidence to support this suggestion would significantly improve the study.**

Due to technical, financial and timing issues, we did not embark in a lipidomic study in the context of the revision of this manuscript; however, in a related project using another *Acetobacter* strain (*Acetobacter* *fabarum*^DsW_054^ from the Newell lab), we have performed an untargeted metabolomics analysis of a culture of this strain in a complete HD. Interestingly, we found a significant production of phospholipids including phosphatidylcholine, phosphatidylethanolamine, and phosphatidylglycerol lipids; an observation that we feel strengthen the hypothesis we are putting forward in our revised manuscript that suggests that Ap^WJL^ may compensate for the lack of choline and its lipid derivatives by producing ethanolamine and/or glycerol phospho-intermediates (pages 23-24, lines 538-546). We prefer at this stage to not mention these unpublished observations in our revised manuscript but we are providing them to the referees as Fig. R2 below.

|  | ***A. fabarum* on complet HD/Sterile complete HD (fold-change)** |
| --- | --- |

| Phosphatidylcholine (PC) | 1,2-dipalmitoyl-GPC (16:0/16:0) | 3,36 |
| --- | --- | --- |
|  | 1-palmitoyl-2-palmitoleoyl-GPC (16:0/16:1)* | 3,92 |
|  | 1-palmitoyl-2-oleoyl-GPC (16:0/18:1) | 30,98 |
|  | 1-palmitoyl-2-arachidonoyl-GPC (16:0/20:4n6) | 0,31 |
|  | 1-palmitoleoyl-2-oleoyl-GPC (16:1/18:1)* | 5,38 |
|  | 1-stearoyl-2-oleoyl-GPC (18:0/18:1) | 30,09 |
|  | 1,2-dioleoyl-GPC (18:1/18:1) | 720,72 |
| Phosphatidylethanolamine (PE) | 1,2-dipalmitoyl-GPE (16:0/16:0)* | 3,71 |
|  | 1-palmitoyl-2-oleoyl-GPE (16:0/18:1) | 253,88 |
|  | 1-palmitoyl-2-linoleoyl-GPE (16:0/18:2) | 5,12 |
|  | 1-stearoyl-2-oleoyl-GPE (18:0/18:1) | 2,44 |
|  | 1,2-dioleoyl-GPE (18:1/18:1) | 197,27 |
| Phosphatidylglycerol (PG) | 1,2-dipalmitoyl-GPG (16:0/16:0) | 7,81 |
|  | 1-palmitoyl-2-oleoyl-GPG (16:0/18:1) | 2,85 |
|  | 1-stearoyl-2-oleoyl-GPG (18:0/18:1) | 1,84 |
|  | 1,2-dioleoyl-GPG (18:1/18:1) | 1,89 |

**Fig. R2 Mass spectrometry measurements of choline and phospholipids.** The table shows the ratio between quantity detected in liquid HD supernatants after 72h growth of *A. fabarum* and quantity detected in sterile HD. Note the accumulation of phospholipids from the phosphatidylethanolamine and phosphatidylglycerol families.

Of note: *Acetobacter* *fabarum*^DsW_054^ (Af) can compensate choline auxotrophy of GF larvae to the same extent as Ap^WJL^ (Fig. R3).

**Fig. R3 *A. fabarum* can compensate choline deficiency.** D50 (*i.e.* day when 50% of the larvae population has entered metamorphosis) of GF larvae and larvae associated with *A. fabarum*^DsW_054^ on HDΔCholine. Boxplots show minimum, maximum and median. GF larvae did not reach pupariation while associated larvae reach metamorphosis on this diet depleted of choline.

**3. Several conclusions are made to state that the bacteria are unable to synthesize a metabolite (e.g. ApWJL is unable to synthesize de-novo choline and pyridoxine). These conclusions are only based on the in silico predictions and should be experimentally verified.**

Using NMR spectroscopy, we confirmed experimentally that Ap^WJL^ does not produce choline in our settings (Related text; page 23, lines 529-532).

Using ultra-sensitive LC/MS technologies, we were able to exclude experimentally the hypothesis that Ap^WJL^ produces pyridoxine (Related text, page 23, lines 534-537). However, our revised genome-based metabolic predictions (Fig. 1B, Table S2; see details below) suggest that Ap^WJL^ may compensate for the lack of pyridoxine by producing intermediates such as pyridoxine-phosphate, pyridoxal-5-phosphate and pyridoxamine. We have added this hypothesis to our revised manuscript (page 23, lines 547-550).

We are affirmative that the pyridoxal 5'-phosphate biosynthesis II pathway, that consists of one single step reaction catalysed by the EC 4.3.3.6, is absent in both bacteria (tblastn run on both genomes gave no significant hit). In the previous version the manuscript, we hypothesized that Lp^NC8^ could produce pyridoxine from phospho-hydroxy-threonine (using a kind of non-conventional derivative route from the pyridoxine biosynthesis I from Erythrose-4-P). We apologize for a misinterpretation of the KEGG map 750 (Vitamin B6 metabolism), that led us to make this assumption. A deeper analysis of the pathway revealed that the pyridoxine 5-phosphate synthase activity (EC 2.6.99.2) is always needed to produce pyridoxine. As this activity is missing in Lp^NC8^, we conclude that the bacteria cannot produce the vitamin. This is true for all of the 54 Lp strains analysed by Martino et al. 2016. We also found that the pyridoxal-5-phosphate synthase activity (EC 1.4.3.5) is present in the genome of Lp^NC8^, indicating that the bacteria are able to switch across different forms of the vitamin’s intermediates depending on the uptake (pyridoxal, pyridoxamine or pyridoxine). Yet, our experimental data (capability of the bacterium to grow on Δpyridoxine diet with no rescue for GF larvae) suggest that pyridoxine is likely not essential for Lp^NC8^. This has now been corrected in the revised Fig. 1B, Table S2 and in the text (pages 10-11, line 223). Concerning Ap^WJL^, the pyridoxine biosynthesis I (from Erythrose-4-P) is almost complete, with the conservation of the key EC 2.6.99.2. The bacterium also possesses the EC 1.4.3.5, allowing the interconversion between the different forms of the vitamin’s intermediates, pyridoxine, pyridoxal or pyridoxamine. This observation is in agreement with our experimental data (optimal growth and rescue of GF-larvae on Δpyridoxine medium), indicating the capability of the bacteria to produce vitamin intermediates but not pyridoxine. Despite our efforts, we were not able to find the enzymes acting at the beginning of the pathway. Several candidate genes can be proposed to complete the two missing steps permitted by the two EC 1.2.1.72 and 1.1.1.29, as many genes in Ap^WJL^ have similar activities (corrections were added in Figure 1B, Table S2 and text (Page 9, line 194-197). Future comparative transcriptomic analyses of Ap^WJL^ grown on complete versus Δpyridoxine media, combined with analysis of mutants are expected to help in discriminating among those candidates Surprisingly, the pyridoxal kinase (2.7.1.35), which allow the production of the phosphorylated forms of pyridoxine or pyridoxal (the active forms of the vitamin), seems to lack in Ap^WJL^, as genome tblastn with homologous proteins found in *Bacillus* and *Acetobacter sp*. (PdxK and PdxY) did not produce any significant hit. Here again, non-conventional kinases may phosphorylate pyridoxal to allow the survival of the bacteria in Δpyridoxine diet. As *D. melanogaster* possesses the pyridoxal kinase, the rescue of GF-larvae can be explained by the sparing of pyridoxal. As this hypothesis is speculative, we decided not to mention it in the revised version of the paper.

Due to technical, financial and timing issues, we did not manage to analyze the pyridoxine biosynthetic pathway intermediates and derivatives using ultra-sensitive LC/MC technologies in the context of the revision of this manuscript. However in a related project using another *Acetobacter* strain (*Acetobacter* *fabarum*^DsW_054^ from the Newell lab), we have performed an untargeted metabolomics analysis of a culture of this strain in a complete HD. Interestingly, we found a significant production of pyridoxamine and pyridoxamine phosphate in this context; an observation that we feel strengthen the hypothesis we are putting forward in our revised manuscript that suggests that Ap^WJL^ may compensate for the lack of pyridoxine by producing intermediates (page 24, lines 547-550). We prefer at this stage to not mention these unpublished observations in our revised manuscript but we are providing them to the referees as Fig. R4 below.

|  | ***A. fabarum* on completed HD/Sterile complete HD (fold-change)** |
| --- | --- |
| pyridoxamine | 5,62 |
| pyridoxamine phosphate | 1,99 |
| Pyridoxate | 1,15 |

**Fig. R4 Mass spectrometry measurements of pyridoxine derivatives.** The table shows the ratio between the quantity detected in liquid HD supernatant after 72h growth of *A. fabarum* and the quantity detected in sterile HD. Note the accumulation of pyridoxamine and to a lesser extent pyridoxamine-phosphate.

**Minor comments:**

**4. Please specify the genotype of Drosophila strain DGRP-RAL-25210**

This is an isogenic wild-type reference strain from the DGRP collection. We have added this information in the methods (page 34, lines 778-779).

**5. Please justify, why did you choose to use y, w as reference line to study auxotrophies (white encodes a tryptophan transporter).**

We thank the referee for raising this issue. There was no specific rationale in using the *yw* strain, this is related to our lab history as we previously worked with this strain to establish bacteria mediated juvenile growth promotion upon chronic undernutrition.

The *white* gene encodes indeed an ABC-type guanine and Trp transporter, which functions as a dimer with the protein Scarlet. However, *w* expression profiling (data available on FlyBase: http://flybase.org/reports/FBgn0003996) indicates no expression in the larval midgut (FlyAtlas data), low to moderate expression in wandering larvae midgut (modENCODE data), low to moderate in the fat-body (FlyAtlas and modENCODE data) but very high in the larvae malpighian tubules (FlyAtlas data). This profile agrees with a function of W as a Trp transporter in malpighian tubules (Sullivan et al. Biochem. Genet. 1980) rather than a transporter of dietary Trp.

To further test the interactions between the *w* genotype and Trp requirements, we tested a w+ line (Bloomington, #35787) on HDΔTrp. We did not find a difference compared to yw larvae: Trp is essential to both GF and Lp^NC8^-associated w+ larvae, and non-essential to Ap^WJL^-associated w+ larvae. We prefer at this stage to not mention these unpublished observations in our revised manuscript but we are providing them to the referees as Fig. R5 below

**Fig. R5 The *w-* allele does not interact with Trp requirements.** D50 (*i.e.* day when 50% of the larvae population has entered metamorphosis) of GF larvae and larvae associated with *A. fabarum* on HDΔTrp. Boxplots show minimum, maximum and median. GF larvae and larvae associated with Lp^NC8^ did not reach pupariation.

---------------

**Reviewer #4:**

**Consuegra et al. use gnotbiotic flies and a chemically defined diet to examine the alleviation of malnutrition by fly gut bacteria. The bacteria convert poor food into more healthy food. While this has been broadly known for quite some time, this study goes into molecular detail as to what specific nutrients are produced and how they influence host health, and to what extent genome annotations are predictive in this case. This is a necessary step towards formulation of precision probiotics, and thus this study provides a template for future work. It puts some real world details to the cartoon view that the microbiome can change our health. I think that is the paper's main strength. We now know from this example how bacteria aid host nutrition based on provision of key nutrients. In some respects this is not novel, but the comprehensive nature of the experiments, using the holidic media for in vitro and in vivo tests, and using the Drosophila model mediates that because it sets up future studies.**

**Overall I think the study design, approach and data are solid and should be published.**

We thank the referee for this positive assessment of our work.

**I have minor comments, mainly to do with the hype, which I found distracting. It would greatly improve the paper to simply focus on the experiments, which are nice. Significantly shortening the Introduction would accomplish that.** **The extensive presentation of the genome annotations in the results section could be condensed or put in the supplement. The experimental validation of the auxotrophies is much clearer**.

The paragraph on aphid nutrition in the introduction has been drastically reduced in response to the reviewer’s comment (pages 6-7, lines 129-141). The descriptions of the metabolic capabilities of the three partners have also been condensed in shorter paragraphs (pages 8-11). We also revised our text to avoid jargon and complex sentences.

**Some additional issues that the authors need to address before publication:**

**There is an issue of whether the effects seen on fly development are due to auxotrophy or due to an overall lack of sufficient macronutrients like protein, fat, and carbohydrate. Auxotrophy means the flies cannot develop without the nutrient. Changes in rate of development are not due to auxotrophy but often a macronutrient limitation, which can be compensated by the bacterial load. The authors find many cases of such compensation. Work from the Ja lab has shown this explicitly, and it should be addressed in the paper (e.g. Yamada et al 2015; Keebaugh et al 2018; Keebaugh et al 2019). Yet while that is a null hypothesis, there certainly can be more precise compensation, which the authors cover to some extent.**

While the Ja lab publications and our lab publication (Storelli et al. 2018) have previously addressed the question of macronutrient compensation by dead microbes, this manuscript reports specific complementation of host auxotrophies by alive bacteria leading to the rescue of host viability while thriving on micronutrient depleted diets. In the revised version of the manuscript (Conclusion section), we have added a new paragraph that makes reference to these previous results, highlights the contribution to the field of the current work and we hope clarifies this point (page 31, lines 714-718).

**To this point, the only additional experiment I would like to see is a measurement of microbial load in the fly food with vs without flies present for each food/bacteria combination where an effect was investigated. This is to address the compensatory effects of having more food due to bacteria. Figure 4C & D have these measurements for two of the conditions. Please expand to cover all the cases where a bacterium can compensate for a missing nutrient. Please address the extent to which microbial load can compensate for the changes in development rate and cite the appropriate literature.**

We understand the referee’s concern and, following his/her suggestions, we have run the requested additional experiments in specific holidic diets set-ups. We assessed the potential correlation between bacterial loads and host growth benefits upon different nutrient drop out in contexts, where both (Full HD,) or each differently (ΔMet HD, ΔPhe HD, ΔNicotinate HD) or only one (ΔCys HD, ΔPyr HD, ΔCholine HD) of the commensal bacteria would impact host growth. These new results are now included in the revised Figures 4B-D and S3, and show that there is no correlation between bacterial load in the larval gut or in the diet and host developmental timing on the tested diets. These results reinforce the notion that commensal bacteria are not “just food” for their animal partner but are biologically active partner to their host. A new paragraph discussing these results is included in the revised version of the manuscript (page 20, lines 460-473).

**References on malnutrition and aphids could be reduced to make room. Specifically regarding the aphid literature, while this is interesting and a classic example of an evolved metabolic interaction, the evidence here is not that flies have such an interaction with their bacterial community. I agree it is likely, but the present experiments do not address it. Maybe future experiments where many different strains were compared for their effect on wild fly development could reveal evolved interactions between the fly and specific strains?**

Indeed, that was not our intention to probe the evolution of metabolic interaction between *Drosophila* and its commensal. The paragraph on aphid nutrition has been drastically reduced in response to the reviewer’s comment (pages 6-7, lines 129-141).

**Figure 4: what does 'Niche Load' mean? Please pick another term. For instance, I suggest 'food alone' in place of 'medium' and 'food + larvae' in place of 'niche load'.**

We apologize for having used our jargon for this. We have now changed to “diet only” or “diet with larvae” in Fig. 5C.

**line 466: In the heat-killed experiments, 10x the number of cells may not be enough to offset bacterial growth on the food. It's only ~3 doublings. So these heat-killed experiments would only reveal compensation for micronutrient deficiencies, which appears to be the case. The requirement for metabolic activity of the bacteria is thus more likely to be based on the bacteria serving as a source of macronutrients -- so this is malnutrition rather than auxotrophy. Rather than doing more experiments, just rephrase this section to note that the bacteria are probably providing macronutrients in the cases where development is still slow. I realize there are some exceptions to this rule, but they're not addressed in this experiment.**

We understand this referee’s concern, but actually bacteria do not significantly increase their load after inoculation on the HD as we use high inoculation titers for testing *Drosophila* developmental timings (10^8^ CFUs for Lp^NC8^ and 10^7^ CFUs for Ap^WJL^ per fly tubes). The bacterial titers in the holidic diets at 6 days post-inoculation reach a plateau at around 10^7^ CFUs and 10^8^ CFUs per tube for Ap^WJL^ and Lp^NC8^, respectively (new Fig. S3B). Hence, in most holidic diets tested bacteria do not grow beyond the conditions used for the HK experiment, in only few specific depleted diets the load increases 10 folds (see new Fig. S3B). Therefore, we believe that our heat-killed bacteria inoculation set-up is an adequate control for both the potential trophic effect of inert bacterial biomass and the contribution of a specific nutrient pool at the start of the experiment in absence of bacterial metabolism.

**Figure 5: The negative controls of the media before microbial growth (0h) need to be shown. Otherwise how can we rule out contamination, which is common in chemical reagents?**

We have now included this information in the legend of Fig. 6 (page 45, lines 1062-1063) and we now provide all the corresponding numerical values in Table S7, which reports the numerical values used to draw all the graphics of the study.

**Also, Figure 5B apppears to be misplotted.**

In fact, this is the correct plot. We were also surprised by the extreme reproducibility of the His amount detected in independent cultures of Lp^NC8^ in ΔHis HD. The exact measurements are the following: 53.08 µM, 52.82 µM and 52.99 µM. They are now included in both revised Fig 6 legend (page 45, lines 1065-1066) and Table S7.

**Lp NC8 was isolated from grass silage and therefore is not a fly commensal; it should not be referred to that way. In the text, please note the origin of the strain and the reason it was selected over a true commensal (presumably the genetic tractability). It does not diminish the study but could mislead readers if they do not know the strain's history.**

We agree with the referee that Lp^NC8^ is not a natural *Drosophila* commensal. However, this strain behaves as a commensal for *Drosophila* in our experimental set-ups, so we are now using the “model commensal” term also for this strain. In order to take into account the referee’s comment, we have added in the Material and Methods section of the revised paper a paragraph specifying the origin of the strains and why they were chosen as model commensal bacteria of *Drosophila*, i.e. they behave as commensals and they are genetically tractable (page 35, lines 797-801).

**The authors refer to in silico analysis but in fact they just ran an automated annotation pipeline and manually checked results. I found that misleading and was waiting for the in silico experiment as I read the paper, but it never came. Computational biologists refer to computational experiments as in silico when an actual experiment has been performed, such as to predict growth rates in FBA models. This is similar to the way experimentalists do something in vitro or in vivo. The results presented here are not an in silico experiment but rather an automated trip to the library. To get rid of this misleading terminology, please change 'in silico' to 'genome annotation' or something like that. For instance, tables S1 and S2 refer to 'genomic analysis', which sounds accurate.**

We agree with the referee that our in silico analysis of metabolism consists of an expert automated pipeline gathering several annotation methods and including a final step of manual curation and comparison with the existing metabolic databases and the literature on the subject. Hence, we replaced the terms “in silico analysis” all along the manuscript by “expert automated annotations”, “genome-based metabolic pathway reconstruction” or equivalent expressions.

**line 28-29 of Abstract: Please remove this sentence. It sounds like a claim for some mystic feature of the microbiome, but as the authors point out in the paper, there are many likely causes, such as incorrect pathway annotations and provision of precursors rather than final products.**

We have now significantly rewritten our abstract in the light of this comment and our latest results.

**Line 32-35 of Abstract: Please remove this sentence. This handwaving about a 'novel facet of the facultative nutritional mutualism' sounds grandiose, but the preceeding sentence covers what the study found. These aspects of the writing are distracting from the nice scientific evidence presented in the results.**

We have now changed the end of our abstract and removed the specific sentence.

**Line 151: If a nutrient can be "functionally compensate[d]" for, it means the organism is not an auxotroph for that nutrient. It is misleading to suggest otherwise. Malnutrition and auxotrophy are different. Please rephrase so that readers don't get the false impression that something more complicated is occurring. The delineation of the 4 cases in the results is good. Please just be careful throughout the manuscript to keep auxotrophy and malnutrition separate.**

We have clarified the notion of “functional compensation” in the conclusion paragraph (page 30, lines 702-713). Accordingly, we have amended our abstract (page 2, lines 27-31) and the end of our introduction (page 7, lines 145-147) to clarify this.

**Line 207-210: Seems somewhat irrelevant. Cut.**

We changed this.

**Line 476: I like this section as it clarifies many of the vagaries in terms of what the microbiome is actually doing.**

Thank you

**Line 571: check typos**

Done

**Line 614: typo: --> "...to directly test this..."**

The change has been made.

**Line 623: typo: --> "accumulation of"**

The change has been made.

**Line 624: typo: --> "except"**

The change has been made.

**In the discussion/conclusion, it would be nice to note to what extent the microbial benefit can be due to catabolism of excess, harmful nutrients as opposed to provision of scarce nutrients.**

This is an interesting area for future research, which is not in the scope of this study as we utilize minimal diets containing no excess or harmful nutrients. Nevertheless, we included a statement in our revised conclusion to cover this exciting perspective (page 31, lines 726-729).

**Methods: Was gap filling (in the pathways) performed? I did not have time to look up the methods paper referenced. It would be worth mentioning it because hypothesized gaps are examined in the results section.**

We did not apply gap filling during or after our metabolic network reconstruction, as these methods are known to produce false positives and need anyway a step of manual curation to correct the gap-filler results (Karp et al., 2018). We then prefer to collect and show evidences about the presence (or absence) of a protein function, integrating results from different annotation methods including KAAS, PRIAM, Blast2GO, and InterProScan (Vellozo et al. 2011), integrating it with a final manual curation step which includes an extensive analysis of the literature on the subject. Indeed, we successfully used this approach for the metabolic pathway reconstruction in other genome annotation projects in the framework of the i5k initiative: IAGC, 2010 and Wilson AC et al. 2010 (pea aphid genome); Baa-Puyoulet et al., 2016 (the ArthropodaCyc Database); Mathers TC et al., 2017 (*Myzus persicae* genome); Panfilio et al., 2019 (*Oncopeltus fasciatus* genome).

[BMC Syst Biol.](https://www.ncbi.nlm.nih.gov/pubmed/29914471)

2018 Jun 19;12(1):73. doi: 10.1186/s12918-018-0593-7.

How accurate is automated gap filling of metabolic models?

[Karp PD](https://www.ncbi.nlm.nih.gov/pubmed/?term=Karp%20PD%5BAuthor%5D&cauthor=true&cauthor_uid=29914471) et al.

[Database (Oxford).](https://www.ncbi.nlm.nih.gov/pubmed/?term=CycADS+AcypiCyc) 2011 Apr 7;2011:bar008. doi: 10.1093/database/bar008. Print 2011.

CycADS: an annotation database system to ease the development and update of BioCyc databases.

[Vellozo AF](https://www.ncbi.nlm.nih.gov/pubmed/?term=Vellozo%20AF%5BAuthor%5D&cauthor=true&cauthor_uid=21474551) et al.

[PLoS Biol.](https://www.ncbi.nlm.nih.gov/pubmed/20186266) 2010 Feb 23;8(2):e1000313. doi: 10.1371/journal.pbio.1000313.

Genome sequence of the pea aphid Acyrthosiphon pisum.

[International Aphid Genomics Consortium](https://www.ncbi.nlm.nih.gov/pubmed/?term=International%20Aphid%20Genomics%20Consortium%5BCorporate%20Author%5D).

[Insect Mol Biol.](https://www.ncbi.nlm.nih.gov/pubmed/20482655) 2010 Mar;19 Suppl 2:249-58. doi: 10.1111/j.1365-2583.2009.00942.x.

Genomic insight into the amino acid relations of the pea aphid, Acyrthosiphon pisum, with its symbiotic bacterium Buchnera aphidicola.

[Wilson AC](https://www.ncbi.nlm.nih.gov/pubmed/?term=Wilson%20AC%5BAuthor%5D&cauthor=true&cauthor_uid=20482655)^1^, [Ashton PD](https://www.ncbi.nlm.nih.gov/pubmed/?term=Ashton%20PD%5BAuthor%5D&cauthor=true&cauthor_uid=20482655), [Calevro F](https://www.ncbi.nlm.nih.gov/pubmed/?term=Calevro%20F%5BAuthor%5D&cauthor=true&cauthor_uid=20482655), [Charles H](https://www.ncbi.nlm.nih.gov/pubmed/?term=Charles%20H%5BAuthor%5D&cauthor=true&cauthor_uid=20482655), [Colella S](https://www.ncbi.nlm.nih.gov/pubmed/?term=Colella%20S%5BAuthor%5D&cauthor=true&cauthor_uid=20482655), [Febvay G](https://www.ncbi.nlm.nih.gov/pubmed/?term=Febvay%20G%5BAuthor%5D&cauthor=true&cauthor_uid=20482655), [Jander G](https://www.ncbi.nlm.nih.gov/pubmed/?term=Jander%20G%5BAuthor%5D&cauthor=true&cauthor_uid=20482655), [Kushlan PF](https://www.ncbi.nlm.nih.gov/pubmed/?term=Kushlan%20PF%5BAuthor%5D&cauthor=true&cauthor_uid=20482655), [Macdonald SJ](https://www.ncbi.nlm.nih.gov/pubmed/?term=Macdonald%20SJ%5BAuthor%5D&cauthor=true&cauthor_uid=20482655), [Schwartz JF](https://www.ncbi.nlm.nih.gov/pubmed/?term=Schwartz%20JF%5BAuthor%5D&cauthor=true&cauthor_uid=20482655), [Thomas GH](https://www.ncbi.nlm.nih.gov/pubmed/?term=Thomas%20GH%5BAuthor%5D&cauthor=true&cauthor_uid=20482655), [Douglas AE](https://www.ncbi.nlm.nih.gov/pubmed/?term=Douglas%20AE%5BAuthor%5D&cauthor=true&cauthor_uid=20482655).

[Genome Biol.](https://www.ncbi.nlm.nih.gov/pubmed/28190401) 2017 Feb 13;18(1):27. doi: 10.1186/s13059-016-1145-3.

Rapid transcriptional plasticity of duplicated gene clusters enables a clonally reproducing aphid to colonise diverse plant species.

[Mathers TC](https://www.ncbi.nlm.nih.gov/pubmed/?term=Mathers%20TC%5BAuthor%5D&cauthor=true&cauthor_uid=28190401) et al.

[Genome Biol.](https://www.ncbi.nlm.nih.gov/pubmed/?term=Molecular+evolutionary+trends+and+feeding+ecology+diversification+in+the+Hemiptera%2C+anchored+by+the+milkweed+bug+genome) 2019 Apr 2;20(1):64. doi: 10.1186/s13059-019-1660-0.

Molecular evolutionary trends and feeding ecology diversification in the Hemiptera, anchored by the milkweed bug genome.

[Panfilio KA](https://www.ncbi.nlm.nih.gov/pubmed/?term=Panfilio%20KA%5BAuthor%5D&cauthor=true&cauthor_uid=30935422) et al

**Line 743: Just a note -- autoclaving sucrose can caramelize it, complicating the chemical composition, and causing batch to batch variation. Best to filter sterilize and add after the autoclaving. But it does not seem to have affected the results here.**

Point taken and indeed in our case we did not detect any event of “caramelization” that would be evidenced by a browning of the solutions.

**Editor comments:**

**As you will read, the reviewers appreciated many aspects of your work. They do raise some concerns, however, that will need to be addressed in a revision.**

**Of particular note, Rev #4 would like to see addressed to which extent the differences in time of development are due to differences in macronutrients availability in the form of bacterial loads. Therefore Rev #4 requests measuring microbial loads in fly food with and without flies in different food/bacteria combinations where an effect was investigated. The academic editor suggests this to be explicitly addressed and discussed in the text.**

**Measuring the bacterial loads in all the conditions and relate them to time of development would be interesting. However, in may be sufficient to measure Acetobacter and Lactobacillus loads in a subset of these.**

We have performed additional experiments to address this point and we now provide these results and discuss them in our revised manuscript. We report the absence of correlation between bacterial load in the larval gut or in the diet and host developmental timing on the tested diets. These results reinforce the notion that commensal bacteria are not “just food” (i.e. a source of macronutrients) for their animal partner but are biologically active partner to their host.

**The Academic Editor also believes that Rev #3's point regarding the discrepancies between predicted and observed auxotrophies/requirements is important to address with further experimental work.**

We provide new experimental data demonstrating that in relevant settings Lp^NC8^ and/or Ap^WJL^ do not synthetize choline, pyridoxine or nicotinic acid.

**S/he also believes that the suggestion for screening to find new genes involved in nicotinate pathway of bacteria is outside the scope of this paper.**

We also share this view. Thank you

**Lastly, the Academic Editor recommends that you reconsider the use of the term "commensal" in the title and throughout the manuscript, using instead “gut-associated bacteria” or, better, “Drosophila-associated bacteria”.**

We believe Lp^NC8^ and Ap^WJL^ behave in our system as model commensal partners of *Drosophila*. Commensalism is either defined as a long-term symbiosis where partner “eat at the same table” (Etymologic definition) or as a long-term symbiosis where one partner gains from the association while the other neither benefit nor is harmed by such association (Biological definition). Lp^NC8^ and Ap^WJL^ association with *Drosophila* is qualified as commensalism under both definitions. Indeed, in our experimental setting Lp^NC8^, Ap^WJL^ and Drosophila remain associated on the long-term even after a single initial inoculation (at least for several days until reaching adulthood), they share the same diet and live in close contact, including within the host’s gut even if this is a transitory state as they are constantly ingested and excreted on the food. Finally, Lp^NC8^ and Ap^WJL^ are beneficial to their host development while their *Drosophila* partner does not alter their growth (actually the host favor the growth of at least Lp in the diet). Given these characteristics, we would like to maintain the use of the term commensal to qualify Lp^NC8^ and Ap^WJL^ in respect to their association with *Drosophila*. Yet we agree that Lp^NC8^, in contrast to Ap^WJL^, is not a natural commensal of *Drosophila* as it has not been originally isolated from flies. We have now clarified this point and stated it clearly in our manuscript by referring to Lp^NC8^ and Ap^WJL^ as “model bacterial strains behaving as commensals” or variants of this statement in multiple occurrence in our text (page 2, lines 19 and 24; Page 4, lines 81-82; page 6, line 124; page 15, line 339; page 30, line 692; page 35, line 800).
